# Application of Artificial Intelligence and Computer Vision for Measuring and Counting Oysters

**DOI:** 10.3390/jimaging11120439

**Published:** 2025-12-10

**Authors:** Julio Antonio Laria Pino, Jesús David Terán Villanueva, Julio Laria Menchaca, Leobardo Garcia Solorio, Salvador Ibarra Martínez, Mirna Patricia Ponce Flores, Aurelio Alejandro Santiago Pineda

**Affiliations:** 1 Facultad de Ingenieria Tampico, Universidad Autonoma de Tamaulipas, Tampico 89336, Mexico; jupino@uat.edu.mx (J.A.L.P.); jdteran@docentes.uat.edu.mx (J.D.T.V.); sibarram@docentes.uat.edu.mx (S.I.M.); mirna_poncef@hotmail.com (M.P.P.F.); aurelio.santiago@uat.edu.mx (A.A.S.P.); 2 Mexican Institute for Research in Sustainable Fisheries and Aquaculture (IMIPAS), Tampico 89370, Mexico; leobardo.garcia@imipas.gob.mx

**Keywords:** computer vision, artificial intelligence, oyster farming farms

## Abstract

One of the most important activities in any oyster farm is the measurement of oyster size; this activity is time-consuming and conducted manually, generally using a caliper, which leads to high measurement variability. This paper proposes a methodology to count and obtain the length and width averages of a sample of oysters from an image, relying on artificial intelligence (AI), which refers to systems capable of learning and decision-making, and computer vision (CV), which enables the extraction of information from digital images. The proposed approach employs the DBScan clustering algorithm, an artificial neural network (ANN), and a random forest classifier to enable automatic oyster classification, counting, and size estimation from images. As a result of the proposed methodology, the speed in measuring the length and width of the oysters was 86.7 times faster than manual measurement. Regarding the counting, the process missed the total count of oysters in two of the ten images. These results demonstrate the feasibility of using the proposed methodology to measure oyster size and count in oyster farms.

## 1. Introduction

Oyster farming is a vital component of aquaculture, valued for its nutritional benefits and its role in enhancing water quality. Advances in seed laboratories and cultivation techniques have heightened global interest.

Various fattening techniques exist worldwide [1], but regardless of the technique used, they all share two typical phases: acclimatization and fattening. Aquaculture considers factors such as water quality and cultivation locations during acclimatization. In the fattening phase, aquaculturists periodically monitor population growth. Then, based on these samples, they measure the time it takes for an oyster seed to reach a commercially usable size. Oyster growth is manually measured and counted using a ruler or vernier caliper. In this process, measurements may vary depending on the individual and the specific method used. It is crucial to automate the measurement of oysters to increase efficiency, uniformity, and data consistency. Kakehi et al. in [2] propose a new method for identifying and counting larvae of the Pacific oyster (Crassostrea gigas). This study aims to automate the traditional manual identification process, which is slow, labor-intensive, and relies on observer expertise. It also utilizes images of the larvae taken under a microscope.

On the other hand, computer vision is a field of computer science that aims to facilitate the interpretation of images, like to human perception. Furthermore, image processing is a branch of computer vision whose primary objective is to identify features within an image to extract information.

Although prior studies have explored the use of deep learning models—such as convolutional neural networks—for automated measurement tasks in aquaculture, these approaches often depend on large training datasets and high-performance computing resources, which restrict their deployment in real-world scenarios. Additionally, many of these models are validated under strictly controlled conditions, limiting their generalizability. There is a lack of lightweight, accessible methodologies that can perform reliably with limited data and moderate hardware. Addressing this gap, our work aims to provide an efficient alternative tailored to field conditions, where computational resources and image volumes may be limited.

In this study, we propose a novel methodology for measuring the averages of length and width of a sample of the American oyster, Crassostrea virginica, utilizing artificial intelligence image processing techniques. This methodology aims to automate the process, improving efficiency and data consistency. Consequently, selecting an appropriate color space that best suits the image’s characteristics is crucial, as it plays a key role in achieving accurate segmentation. Moreover, the positioning of the oysters in the image and the quality and standardization of the photographic field during image capture are essential factors for obtaining satisfactory results in this investigation. Proper alignment and uniform lighting can significantly impact the precision of the automated measurements, ensuring reliable and consistent outcomes across different samples.

The main contributions of this study are summarized as follows:

- We propose a methodology based on a modified DBSCAN, an artificial neural network, and a random forest model to segment and clean oyster images in field conditions.
- We develop a comprehensive measurement pipeline that calculates oyster dimensions based on real-world references, enabling automation without the need for manual calibration tools.
- We evaluate the methodology on 250 field images of *Crassostrea virginica*, reporting effective segmentation, noise filtering, and dimensional estimation under varying image conditions.

The remainder of this paper is organized as follows: Section 3 contains the proposed methodology, Section 4 contains the results obtained, Section 5 contains a discussion, and Section 6 contains the conclusions of this paper.

## 2. Related Works

Several studies in aquaculture and related fields have applied artificial intelligence and image processing techniques to automate measurement and classification tasks. For instance, Yang et al. [3] used appearance-based deep learning methods such as KNN, ANN, and Random Forest to identify fish in controlled aquaculture environments based on texture, shape, and color recognition.

Machine learning algorithms have also proven effective in more complex environments. Kumar et al. [4] present a disease diagnosis system for fish using multispectral images captured under real-world conditions. They apply machine learning algorithms, such as SVM, Random Forest, and KNN, to analyze tissues affected by common diseases, including aeromoniasis, columnaris, and epizootic ulcerative syndrome (EUS), achieving more than 95% classification accuracy. Our work shares similarities with this study in its focus on automating the visual analysis of aquatic organisms under real cultivation conditions.

Furthermore, Xue et al. [5] employed artificial intelligence methods, such as multilayer perceptrons and neural networks, to predict traits that typically require the sacrifice of fish. This less invasive technique reduces the number of traditionally performed invasive measurements, thereby minimizing the impact on fish health.

In line with these non-invasive approaches, Correia et al. [6] present a fish length estimation system based on RGB images, employing deep learning architectures. Their study emphasizes that precise segmentation is crucial to avoid measurement errors, particularly when distracting elements or cluttered backgrounds are present.

Similarly, Naseer et al. [7] propose a multi-task neural network that integrates fish detection and classification in noisy underwater environments. Their architecture includes spatial and channel attention modules to enhance the extraction of relevant visual features. Despite poor lighting and occlusions, the model achieves high accuracy in both species identification and organism localization, supporting the need for robust segmentation and detection models adapted to uncontrolled conditions.

Also relevant is the work of Zhao et al. [8], who develop an automated fish measurement system based on computer vision, combining Mask R-CNN segmentation and geometric processing techniques to estimate lengths with high precision. Their approach accounts for variations in lighting and positioning using standard images, reinforcing the feasibility of applying robust segmentation models in real-world environments.

In addition to measurement tasks, computer vision techniques have also been applied to counting in aquaculture. For instance, Andini et al. [9] present an automated system for counting fish fry based on digital image processing. The authors employ a pipeline of preprocessing, binary segmentation, and noise filtering to detect and count individual organisms with high accuracy.

In the energy domain, Macabebe et al. [10] compare the performance of K-Means and DBSCAN clustering to identify hotspots in photovoltaic panels. Their study highlights the robustness of DBSCAN in segmenting data with irregular boundaries, which inspired us to adapt it for oyster segmentation under variable lighting and field conditions. Their system was validated under real aquaculture conditions, demonstrating reliable performance in uncontrolled environments. This study further confirms the effectiveness of computer vision for automatic analysis in aquaculture.

Regarding oyster-specific applications, Han et. al. [11] develop a genetic map in oysters to identify quantitative trait locus (QTL) associated with shell orange color and sex. This study demonstrates that the HSV color space enables a more accurate characterization of color variation in oyster shells. Unlike the RGB model, the HSV model separates chromaticity into the hue (H) component, which enhances segmentation results by providing more precise color distinctions. This separation allows the algorithm to focus on hue value for more effective feature extraction, minimizing the influence of lighting variations or intensity that may affect the RGB color space. For this reason, we use the HSV color space in this article, where contrasting the color of oysters against the background is essential.

Other works also utilize HSV for segmentation tasks. For instance, Khanal et al., Sugimoto et al., and Giuliani et al. [12,13,14] demonstrate the advantages of HSV over RGB in segmenting agricultural and biological images. However, most of these studies ultimately visualize results in RGB for interpretability.

In segmentation, Loke et al. [15] applied machine learning techniques to perform segmentation into superpixel groups. For this purpose, they constructed a new model called F-DBScan, based on the DBScan algorithm, which limits the search area to (W × H)/N, where W and H are the dimensions of the image and N is the number of superpixels. An image in the CIELAB color scale separates luminosity and colors, allowing for greater efficiency in the segmentation process. This new method is six times faster than the previous methods, maintaining competitive segmentation quality and noise resistance.

Finally, Loke et al. [16] discussed the use of the Circle Hough Transform (CTH) in HSV space for detecting circular references. Their findings confirm that CTH is effective in controlled settings but may fail when multiple similar-color circles are present. We considered this limitation when designing the reference marker in our experimental setup.

## 3. Materials and Methods

**Figure 1:**
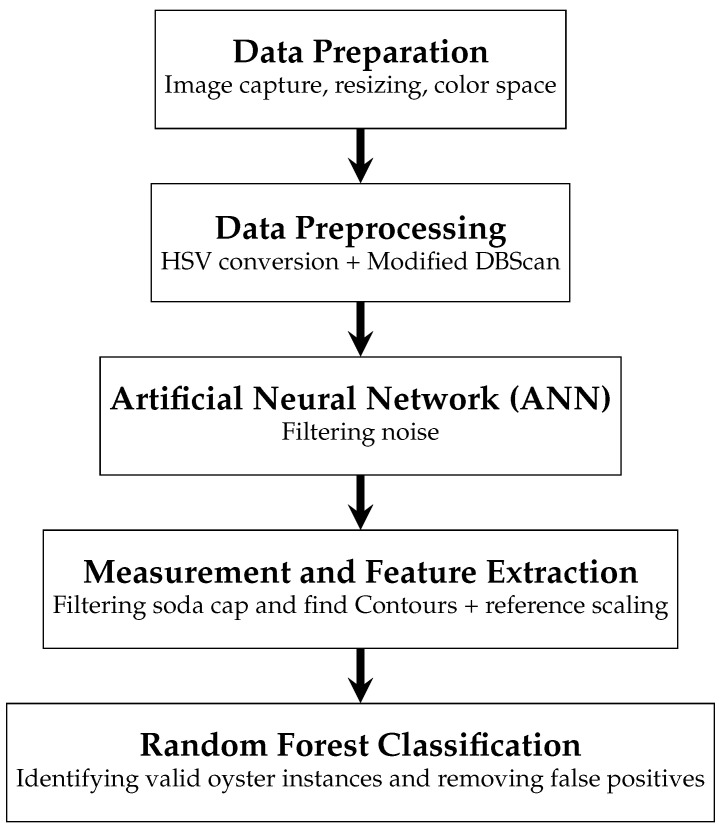
Proposed methodology.

Figure 1 shows the methodological structure used. The 250 images were filtered and resized using OpenCV. Our methodology consists of five main stages.

First, we captured the images. Next, we resized them by dividing both height and width by four to speed up processing. Then, we converted the resized images to the HSV color space, which allows for better separation of brightness from color information. This resizing step was essential for segmentation and later cleaning stages, as the process treats the image as a matrix of pixels. Maintaining the most relevant visual features while reducing resolution helps optimize processing time without sacrificing accuracy. We used OpenCV for all image-handling tasks due to its efficiency and broad functionality [17,18,19].

Subsequently, we applied a modified Density-Based Spatial Clustering of Applications with Noise (DBScan) algorithm. This clustering technique identifies each oyster separately from the background and generates groups. We selected this clustering technique over convolutional models such as CNNs, primarily due to the limited dataset size, which was insufficient for proper deep learning training. Moreover, the relatively regular spatial distribution of the oysters made DBScan a more suitable and computationally efficient option for this task.

Once clustering was completed, we proceeded to clean the identified groups by removing components that did not correspond to oysters using an artificial neural network (ANN). These include shadows or the soda cap used as a reference object. The ANN filters out these non-relevant elements, ensuring that only valid candidate regions remain for measurement.

Following this cleaning step, we utilized the Find Contour and Circle Hough Transform (CTH) functions from the OpenCV framework to define the oysters’ contours and identify the diameter of the cap, respectively. With this reference, we estimated the number of pixels per millimeter, enabling the calculation of each oyster’s dimensions (length and width) in physical units.

Despite these preprocessing steps, our results may still contain noise (small groups of pixels that are not oysters) and instances of fused oysters (two or more oysters trapped in the same group of pixels). To address this issue, we apply a Random Forest model. This Random Forest identifies small groups of pixels as noise and discards them; additionally, it identifies fused oysters and halves them through the longest dimension, whether in width or height. This step is crucial in ensuring the accuracy of our oyster analysis.

### 3.1. Data Preparation

**Figure 2:**
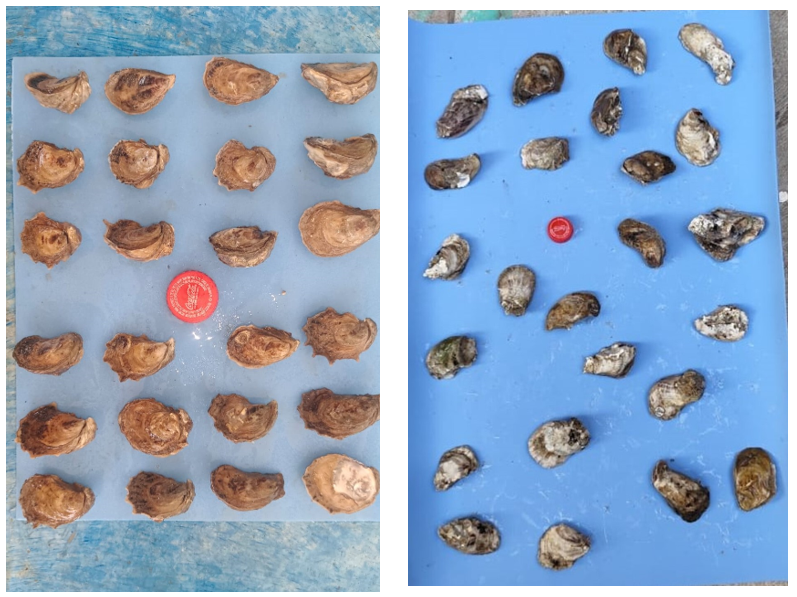
Example of the used images.

We obtained 250 field images, as shown in Figure 2, where oysters are evenly distributed on a blue background. We obtained the blue background by placing foam paper behind the oysters, which made the segmentation process more precise. The maximum dimension of the images is 1300 × 1300 pixels. We captured the images using a standard smartphone camera from the Redmi Note 13 Pro, which features a 200 MP-wide sensor with an aperture of f/1.7. All images were taken specifically during period of time between mid-morning and late afternoon (approximately 10:00 a.m. to 4:00 p.m.) to ensure consistent lighting conditions and avoid shadows caused by low sun angles. We did not use flash, and the smartphone was placed perpendicularly above the oyster sample, at a fixed height of approximately 40 cm. Each image contains a soda cap as a reference point, due to its widespread availability. These acquisition settings are recommended to ensure consistent segmentation quality and model performance under real field conditions. We have a limited number of images because there are few reliable, clean, and tested images of Crassostrea virginica oysters available.

We divided the 250 images into two groups: the first group, comprising 200 images for training, and the second group, comprising 50 images for testing the methodology. From the train group images, we manually measure the height and width of the oysters and the cap.

Selecting the most appropriate color space is essential for image processing. Generally, the Red, Green, and Blue (RGB) color space is used mostly to visualize images. However, the distance between two color points differs due to differences in vision characteristics, resulting in disadvantages in obtaining the three-color characteristics (hue, luminance, and saturation) through RGB data [20]. For this reason, in our case, we use the Hue, Saturation, and Value (HSV) color space, as it separates the brightness information from the image, allowing us to work directly with color values.

### 3.2. Data Preprocessing

**Figure 3:**
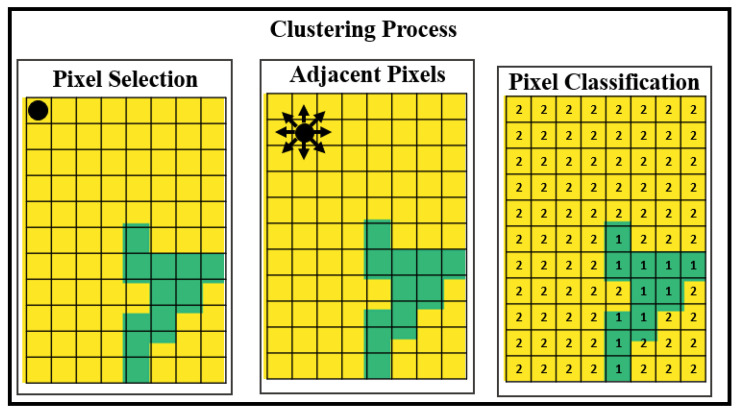
DBScan algorithm for image segmentation.

We applied a modified DBScan (Density-Based Spatial Clustering of Applications with Noise) algorithm to segment the oysters and separate them from the image background. In our modified DBScan, we calculated the distance between all points and the eight closest pixels. The epsilon value for density estimation is dynamically calculated based on the image’s average hue (H) value in the HSV color space. Additionally, we exclude pixels from the edges of the image. This process is shown in Figure 3.

We classified each pixel of the image into one of three types: a value of −1 is assigned to pixels identified as outliers or as located on the edge of the image, 1 is assigned to pixels representing the boundary elements of detected groups, and 2 is assigned to pixels representing the centroids of detected groups within our modified DBScan algorithm.

Furthermore, once the modified DBScan identifies the pixel groups, we discard those with more than one-tenth of the total number of pixels in the image, as they should represent the background and should not be considered for counting or measurement.

### 3.3. AI Techniques

#### 3.3.1. ANN

It is important to note that the ANN is applied after the DBSCAN segmentation stage. These techniques are not combined, but are used sequentially within the methodology. We used an artificial neural network (ANN) to remove noise and filter out groups of pixels that do not correspond to oysters. This noise typically includes oyster shadows, small pieces of rubbish typical of the environment, and areas that differ in color from the background and are not large enough to be an oyster. The ANN also filters out the reference object (soda cap), which is irrelevant for biological analysis. We used 200 images to train the ANN, resulting in 15,095 pixel groups. The neural network design includes one hidden layer containing 100 neurons with a ReLU activation function. We train the model for 200 iterations using the Adam optimizer and categorical cross-entropy as the loss function. We chose this configuration based on preliminary experimentation.

The dataset used to train the network is summarized in Table 1, which lists the attributes for each group. Each row represents a group of pixels with its associated features.

**Figure 4:**
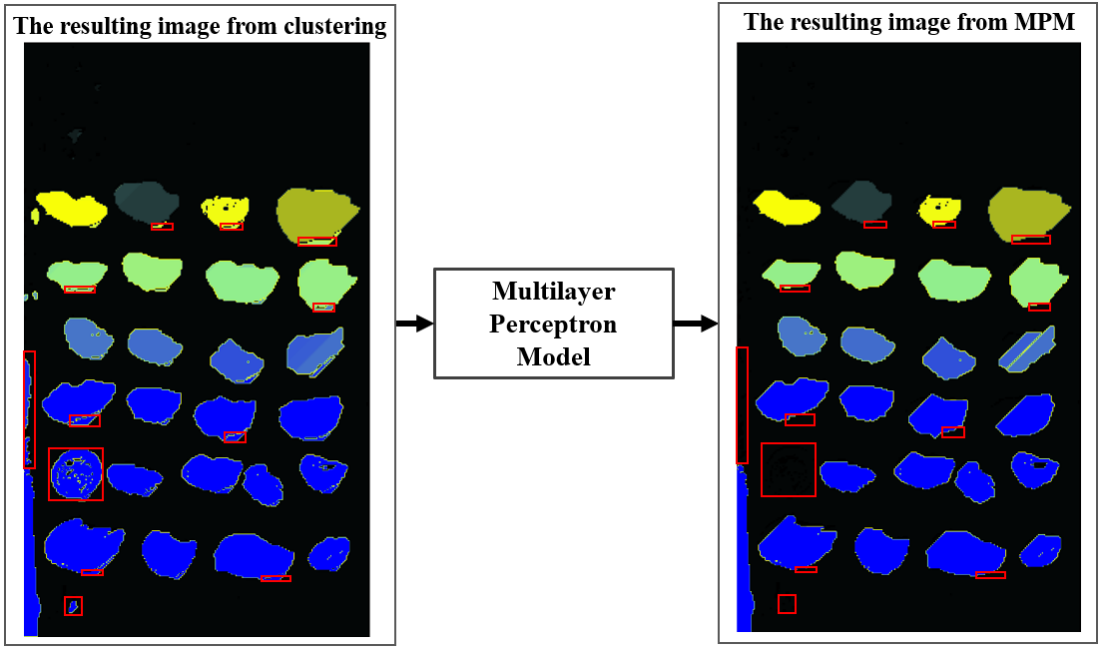
Application of the artificial neural network.

The %mean attribute corresponds to the percentage of pixels in that group relative to the total pixels of the image containing that group, and the %std. dev attribute is the standard deviation of the %mean attribute. The %pixels attribute corresponds to the percentage of pixels in the group based on the total percentage of pixels in the image. The class attribute is zero if the group corresponds to oyster shadow or background noise, one to an oyster, and two if the group of pixels includes two overlapping oysters or an oyster with overlapped noise. The result of applying this neural network is depicted in Figure 4, demonstrating the noise-cleaning of the oyster groups. We used the remaining 50 images to validate the neural network.

#### 3.3.2. Measurement and Feature Extraction

After filtering the segmented regions using the neural network, we extracted geometric features from each remaining group to estimate their physical dimensions. We first applied the findContours function from the OpenCV library to detect the external rectangular boundaries of each group. This step was performed on binarized images to enhance contour detection accuracy and reduce false boundaries.

To determine the measurement scale in millimeters, we used the soda cap present in each image as a reference. Specifically, we employed the Circular Hough Transform (CTH) function from OpenCV with the original image in the YUV color space. We chose this color space because it provided better performance in identifying the circular contour of the cap compared to RGB or HSV representations.

Once we know the diameter of the reference object, we convert the number of pixels in each group to a real-world scale in millimeters. Using the contours previously identified, we measured each group’s bounding box in pixels to determine its width and length. Then scaled these values using the known diameter of the cap. For each group, we also extracted the coordinates of its upper-left (p1X, p1Y) and lower-right (p2X, p2Y) bounding points.

The resulting feature set, comprising scaled length, width, and positional attributes, was then used in the next stage for classification using a Random Forest model to further refine the oyster identification process and address cases of overlapping or noisy groups.

#### 3.3.3. Random Forest

After measuring each group’s length and width using the bounding contours and the reference scale, I applied a Random Forest classifier to refine the identification of valid oyster regions. This additional classification step aimed to eliminate residual noise, such as overlapping oysters, complex artifacts, or irregularly segmented structures that may persist.

We trained the Random Forest model using a dataset of 1015 groups from 200 labeled images. Based on preliminary experimentation, configured the model with 100 decision trees, balancing accuracy with computational efficiency, and set a fixed random state of 42 to ensure reproducibility. Table 2 shows the features extracted for each group. We use accuracy as the primary performance metric and compute the confusion matrix to evaluate classification quality across all classes.

The %large attribute represents the group’s length and width in millimeters. We divided these values by half the total image size and then multiplied by 1000. We used the x and y coordinates of the upper-left point to define %p1X and %p1Y, and the lower-right point to define %p2X and %p2Y.

We set the class attribute as zero for noise, one for an oyster, and two for two joined oysters or an overlapped oyster. For class 2 groups, we halved the measured length to reduce their impact on the average.

## 4. Results

In this section, we show the results of our proposal. We carried out the computational experimentation on a computer with a Processor Intel(R) Core(TM) i7-9750H CPU @ 2.60 GHz 2.59 GHz, a RAM of 16 GB, operating system of 64 bits, Windows 11 Home Single Language, 23H2 Version, 22631.4169 OS Compilation, and a Windows Feature Experience Pack 1000.22700.1034.0.

**Figure 5:**
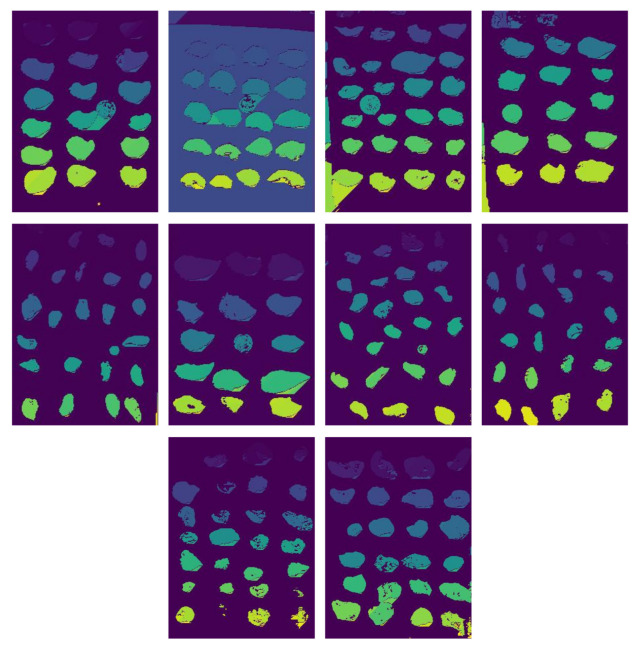
Result of segmentation with variation in DBScan.

Figure 5 shows ten of the fifty resulting images after the segmentation process using the modified DBScan. We applied this segmentation to each of the ten images used to evaluate the methodology. We observe that in most cases, oysters are efficiently identified. However, the algorithm also identifies other groups corresponding to the background as individual groups, depending on the image quality or the presence of objects unrelated to oysters.

**Figure 6:**
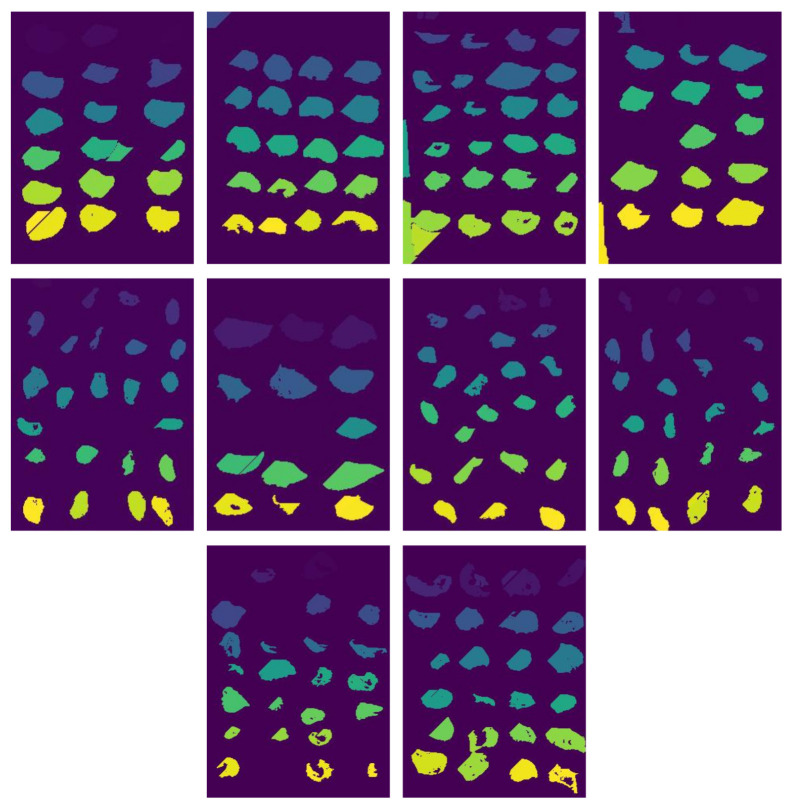
Result of cleaning proccess with ANN.

After segmentation, we observed the need for an additional cleaning step to remove minor errors, such as shadows of oysters and elements unrelated to oysters that may appear in the image. To address this, we applied for an artificial neural network (ANN), trained to filter out such non-oyster components from the segmented groups. In Figure 6, we can see that this cleaning process, performed by the ANN, significantly improves the overall quality of the segmentation in the same ten images used before. Based on experiments, an average of 7000 pixels in each image were modified or removed because they did not match the oysters.

**Figure 7:**
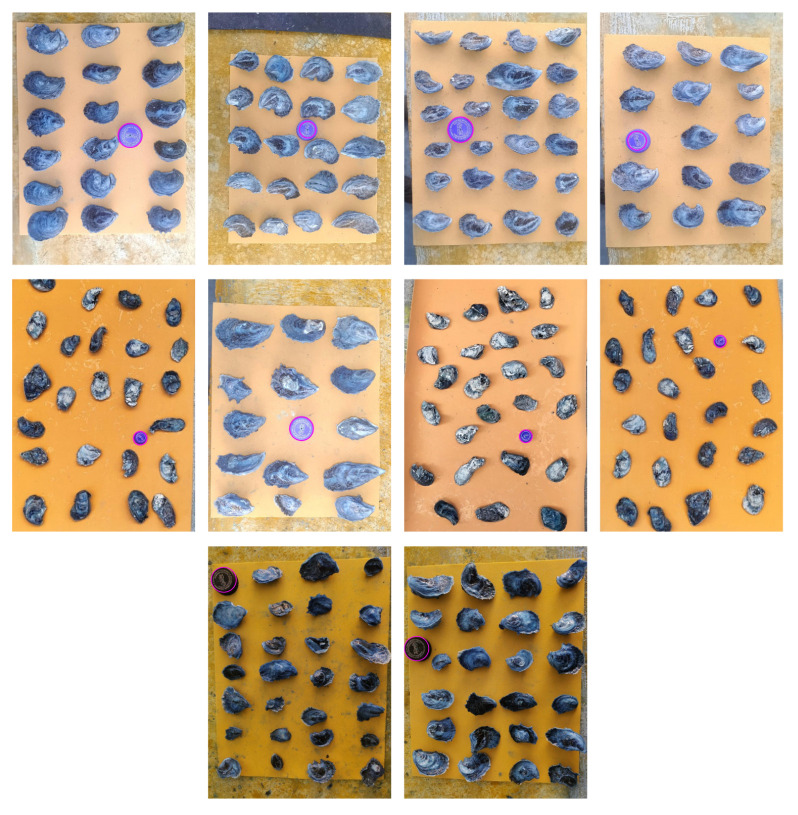
Results of ref (cap) identification.

We use the contours found in the segmented and cleaned image to measure each oyster, which has been processed with the ANN model. For contour identification, we used the OpenCV framework to process the image. Additionally, we requested the reference (cap) dimensions for measurement. The lid is identified as a circular object, as illustrated in Figure 7.

**Figure 8:**
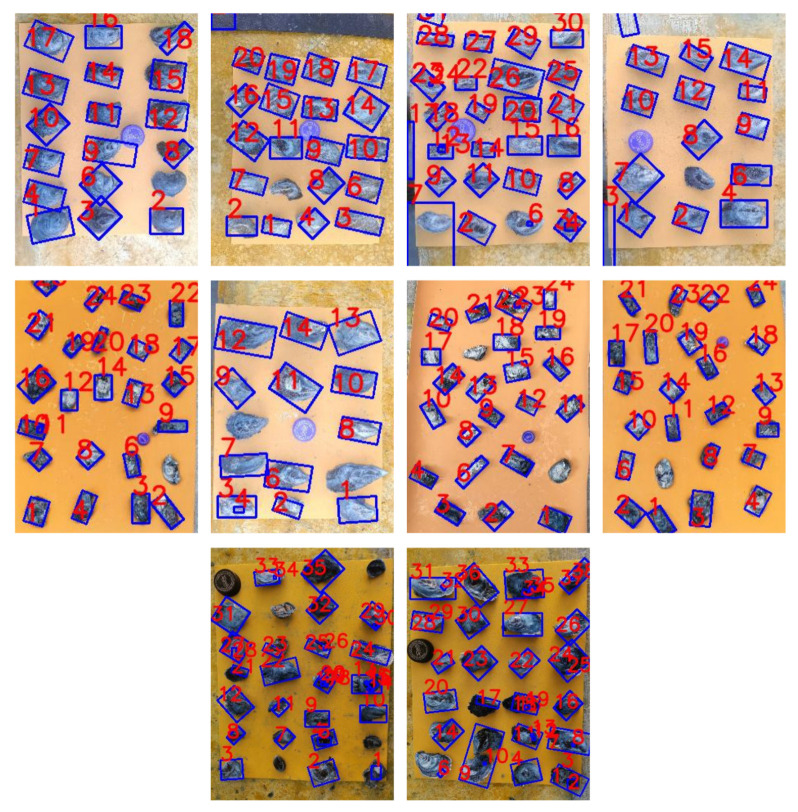
Result of cleaning proccess with Random Forest model.

Figure 8 highlights the contours of each oyster in ten of the 50 images used for the evaluation after cleaning with the Random Forest model. We outlined the oysters correctly classified in blue. We mark the group of pixels we removed in black. Finally, we highlighted in green the contours of two stuck oysters, which we split into two equal parts based on their length.

We manually measure the length and width of oysters in ten images using a caliper. Four people perform this process to estimate measurement error. The oysters are also measured using the proposed method. We also record the process time and oyster count per image.

To validate the stability of the proposed methodology, we then conducted a statistical test on 14 images containing the same 21 oysters arranged in various forms and positions. This assessment used a Wilcoxon test for two paired samples.

For these 14 images, one person measured them all manually with a caliper. However, we cannot consider manual measurements to be accurate values because they are measured differently, even when taken by the same person. Therefore, we compared the standard deviation of the 21 oysters, arranged randomly, as measured by a person, with that obtained using the proposed methodology. For this reason, we conducted a Wilcoxon test for two paired samples. Table 3 and Table 4 present the Wilcoxon test results, comparing the measurements’ standard deviation across the 14 images for the length and width dimensions, respectively.

The tables show that the measurements taken by the system exhibit lower variance than the manual measurements of the same oysters because the negative ranks have a higher average than the positive ones. These results indicate that the system’s measurements are statistically more consistent than those obtained manually. Regarding the variances in width, the statistical test shows that both measurements are statistically equivalent, as the *p*-value is greater than 0.05, which is the recommended criterion in this case for determining comparability.

For the final methodology evaluation, we conducted an experiment to test the robustness and generalizability of our approach. We used 250 images, training on 20 and testing on the remaining 230. This setup tested model performance with minimal training data.

**Figure 9:**
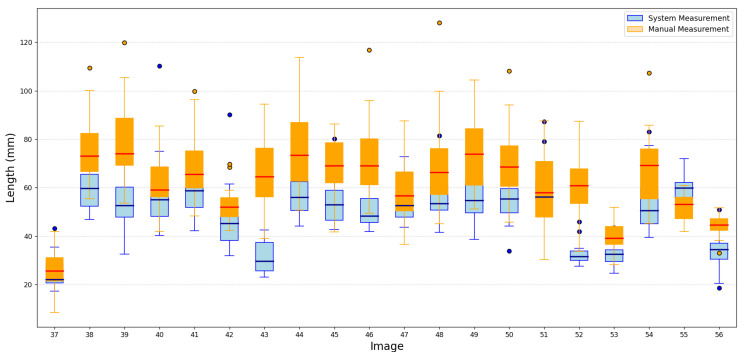
Portion results of length images.

**Figure 10:**
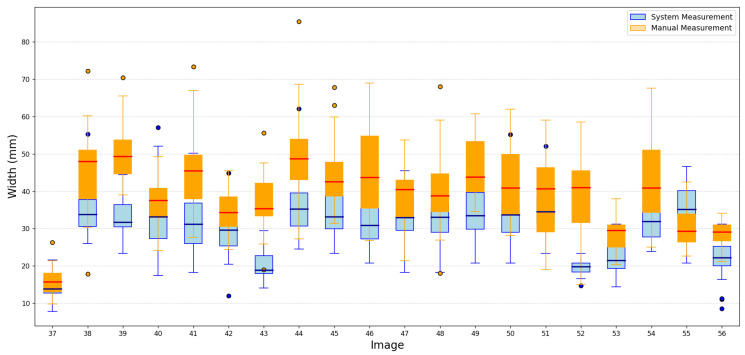
Portion results of width images.

Despite the small training set, the results showed that the proposed methodology maintained reliable performance, demonstrating its potential to generalize effectively. A portion of these results is shown in Figure 9 and Figure 10.

The proposed system provides a robust and efficient solution for image-based measurement tasks, offering both precision and significant time savings. To quantify its accuracy, we calculated the Mean Absolute Error (MAE) between the system measurements and the manual measurements for the 230 test images, considering both length and width. The MAE obtained was 12.2 mm for length and 7.8 mm for width. These results confirm that the proposed approach achieves a reasonable level of precision, even under constrained training conditions, and can be considered a robust solution for measurement tasks based on image analysis. Additionally, the average processing time per image using the system is approximately one minute, whereas manual measurement by a person takes around two minutes per image. This time represents a significant reduction in processing time, further supporting the practical applicability of the proposed system.

## 5. Discussion

**Figure 11:**
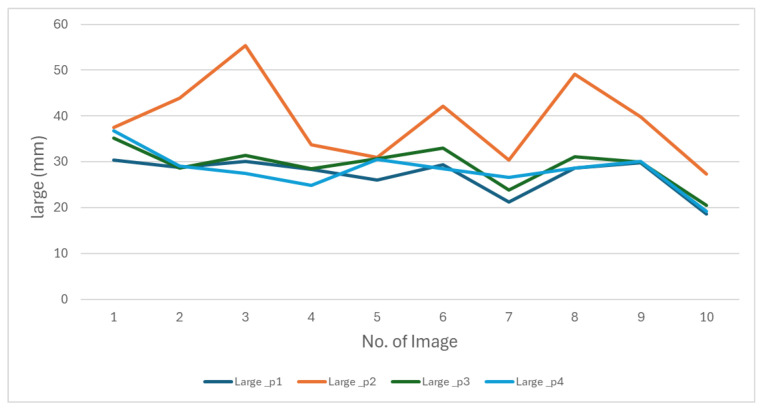
The behavior of oyster length measurements made by four people manually.

**Figure 12:**
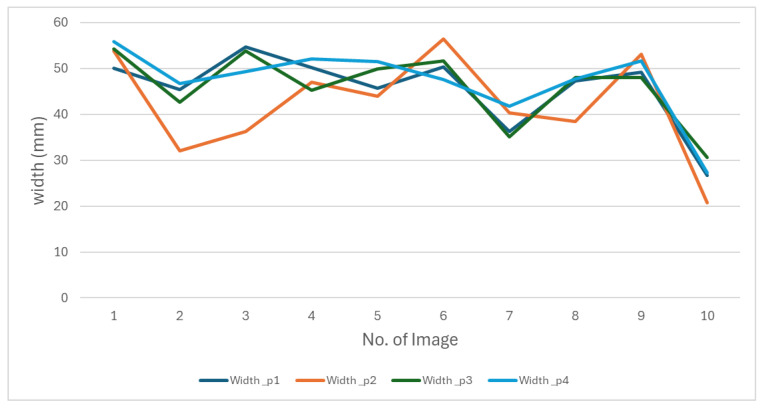
The behavior of oyster width measurements made by four people manually.

We conducted an evaluation in which four individuals manually measured the same 50 images. We presented the results of measurements of the length and width of this evaluation in Figure 11 and Figure 12.

The figures show that the behavior of the measurements varies depending on the person performing them. These differences reflect the subjectivity inherent in manual measurements, as everyone applies different criteria to determine length and width. This variability makes it impossible to establish a single measurement as an absolute reference. These results highlight the need to develop a methodology that produces consistent data.

Additionally, the average manual measurement time for the oysters in each image was 5 min per image, including creating the final report for each measurement. In contrast, the proposed methodology completed the same task in 40 s using only CPU resources, without GPU acceleration. All tests were conducted on a mid-range office laptop (Intel Core i7-9750H, 16 GB RAM), confirming that the methodology is computationally efficient and feasible for near real-time applications.

**Figure 13:**
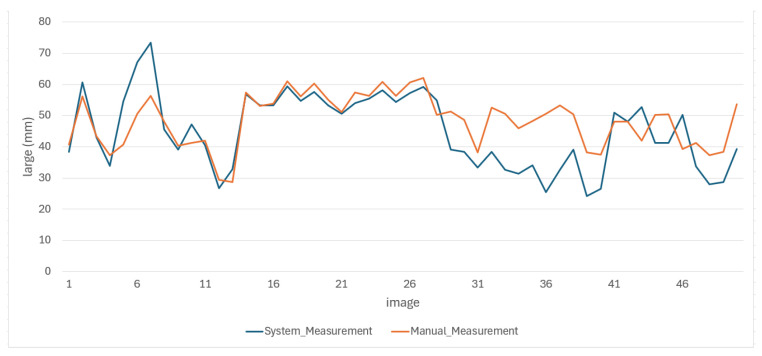
Behavior of oyster length measurements made by both methods.

**Figure 14:**
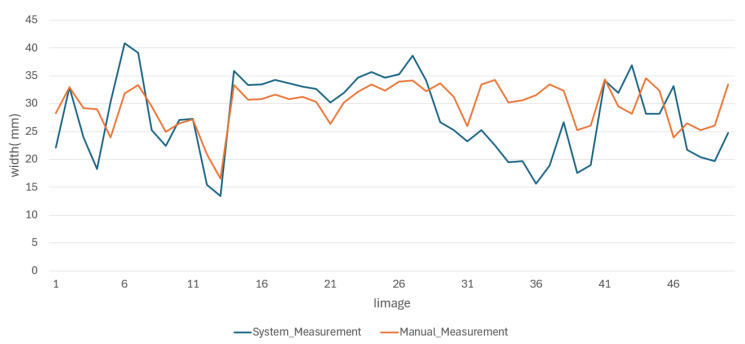
Behavior of oyster width measurements made by both methods.

The results of the manual measurements, along with their respective errors, and the measurements made using the proposed methodology for the length and width of the oysters are presented in Figure 13 and Figure 14, respectively.

From the behavior of the measurements in both figures, it is clear that the average results obtained through the proposed methodology for the length and width of the oysters are remarkably similar to those obtained through manual measurements. These results are either very close or fall within the error range of manual measurements, providing a strong reassurance of the methodology’s accuracy. This consistency underscores the reliability of the proposed methodology. We obtained good results due to the standardized conditions in position and background during the image capture of the 50 test images, which minimized external variability between images. In the future, we do not expect significant changes to affect image capture, as maintaining these conditions is a requirement for both this research and local farmers.

On the other hand, unlike the manual counting method, the proposed methodology yields an error of two oysters in the count. The discrepancy in oyster counts is due to the fact that the missing oysters are significantly darker or dirtier than those in the image. Nevertheless, even if the counting misses a couple of oysters, the objective of this study is to measure the average length and width of a sample of oysters; therefore, even if the proposed methodology misses a couple of oysters, it will not affect the average length and width of the sample.

## 6. Conclusions

This work develops a method for measuring the average length and width of American oysters (Crassostrea virginica). It also counts oyster samples using AI and computer vision on images from any mobile device.

Unlike the traditional manual measurement carried out in oyster farms, the proposed methodology was 86.7 times faster in measuring the length and width of the oysters. We attributed the minor error observed in the measurement to the increased precision in the measurement methodology, given that it operates on a pixel scale.

The proposed method integrates artificial intelligence components, such as an artificial neural network (ANN) for filtering non-oyster elements and a Random Forest model for identifying valid oyster instances. These AI techniques significantly contribute to the robustness, scalability, and full automation of the measurement process.

These results demonstrate the feasibility of using the proposed methodology in oyster farming and its potential for other applications. As a digital processing technique, it is highly efficient in terms of speed, stability, and consistency in measuring the size and quantity of oysters in each image, which enhances the efficiency of this process in oyster farming. In contrast to more complex approaches based on convolutional neural networks, which typically require extensive annotated datasets and GPU-accelerated environments, our methodology offers a lightweight and practical alternative. It delivers reliable measurements using only 250 field images and computationally inexpensive models, such as DBSCAN, MLP, and Random Forest. These components run entirely on standard office hardware without sacrificing precision. This design choice closes a notable gap in the literature by providing an accessible and efficient method for oyster measurement and counting that can be deployed directly in real-world aquaculture settings. Thus, this work contributes a novel, low-cost, and scalable solution that does not depend on high-end computing resources or massive training datasets, making it suitable for adoption in low-infrastructure environments.

As future work, we consider it relevant to apply this methodology to other aquaculture crops, opening new possibilities for the industry.

## Figures and Tables

**Table 1:** Dataset for train ANN.

No.	% Mean	% Std. Dev.	% Pixels	%Class
1	0.4	0.14	0.8	0
2	0.4	0.14	1.23	1
3	0.4	0.14	0.30	2
4	0.4	0.14	0.03	0
.	.	.	.	.
.	.	.	.	.
.	.	.	.	.
15,095	0.19	0.46	0.60	1

**Table 2:** Dataset for training Random Forest.

No.	%Large	%Width	%p1X	%p1Y	%p2X	%p2Y	Class
1	0.75	0.43	0.27	0.78	0.43	0.74	1
2	0.36	0.24	0.07	0.91	0.14	0.86	0
3	2.48	0.98	0.58	0.51	0.98	0.61	2
4	1.16	0.73	0.25	0.56	0.44	0.63	1
.	.	.	.	.	.	.	.
.	.	.	.	.	.	.	.
.	.	.	.	.	.	.	.
1015	0.19	0.46	0.60	1	0	0	0

**Table 3:** Wilcoxon test results for length.

Comparison	N	Mean Rank	Sum of Ranks
Negative Ranks (Length_S < Length_M)	15	11.07	166.00
Positive Ranks (Length_S > Length_M)	5	8.80	44.00
Ties (Length_S = Length_M)	1	-	-
**Statistic**	**Value**		
Z	−2.28		
Asymptotic Sig. (2-tailed)	0.023		

**Table 4:** Wilcoxon test results for width.

Comparison	N	Mean Rank	Sum of Ranks
Negative Ranks (Width_S < Width_M)	13	8.23	107.00
Positive Ranks (Width_S > Width_M)	8	15.50	124.00
Ties (Width_S = Width_M)	0	-	-
**Statistic**	**Value**		
Z	−0.30		
Asymptotic Sig. (2-tailed)	0.768		

## Data Availability

The data supporting the findings of this study are available from the corresponding author upon reasonable request.
